# Narratives of sex workers: intimate partner violence and coping strategies

**DOI:** 10.1590/0034-7167-2024-0180

**Published:** 2024-12-16

**Authors:** Milena Oliveira Costa, Valéria Cristina Christello Coimbra, Michele Mandagará de Oliveira, Clarissa de Souza Cardoso, Vania Dias Cruz, Ariane da Cruz Guedes, Josiane da Costa Moreira, Luciano Santos Gentilini

**Affiliations:** I Universidade Federal de Pelotas. Pelotas, Rio Grande do Sul, Brazil; II Universidade Federal do Pampa. Uruguaiana, Rio Grande do Sul, Brazil

**Keywords:** Sex Workers, Intimate Partner Violence, Women, Personal Narrative, Coping Strategies., Trabajadores Sexuales, Violencia de Pareja, Mujeres, Narrativa Personal, Estrategias de Afrontamiento.

## Abstract

**Objectives:**

to understand the narratives of sex workers about violence suffered by intimate partners and their coping strategies.

**Methods:**

qualitative research, focused on thematic oral history, carried out with six sex workers in southern Brazil, who responded to in-depth interviews using a flexible script. Thematic content analysis was used.

**Results:**

the study included cisgender women who self-identified as half black and half white. Most had children and were separated. They reported abusive relationships by their intimate partners, with emphasis on verbal, psychological, financial, and physical violence and attempted femicide. Such violence resulted in coping strategies, such as avoiding emotional bonds and maintaining a discreet life.

**Final Considerations:**

intimate partner violence is prevalent among participants, leading them to adopt strategies to preserve their safety and well-being, highlighting the need for public policies that meet their particularities and guarantee protection.

## RESUMEN

**Objetivos:**

comprender las narrativas de trabajadoras sexuales sobre la violencia sufrida por sus parejas íntimas y sus estrategias de afrontamiento.

**Métodos:**

investigación cualitativa, centrada en la historia oral temática, realizada con seis trabajadoras sexuales del sur de Brasil, quienes respondieron a entrevistas en profundidad utilizando un guión flexible. Se utilizó análisis de contenido temático.

**Resultados:**

en el estudio, participaron mujeres cisgénero que se declararon mitad negras y mitad blancas. La mayoría tuvo hijos y fueron separados. Denunciaron relaciones abusivas por parte de sus parejas íntimas, destacando violencia verbal, psicológica, patrimonial y física e intento de feminicidio. Dicha violencia resultó en estrategias de afrontamiento, como evitar vínculos emocionales y mantener una vida discreta.

**Consideraciones Finales:**

la violencia de pareja prevalece entre los participantes, lo que los lleva a adoptar estrategias para preservar su seguridad y bienestar, destacando la necesidad de políticas públicas que atiendan a sus particularidades y garanticen protección.

## INTRODUCTION

The United Nations defines violence against women as “any act of gender-based violence that results in, or is likely to result in, physical, sexual, or mental harm or suffering to women, including threats of such acts, coercion or arbitrary deprivation of liberty, whether occurring in public or in private life”^(1)^.

Intimate partner violence (IPV), in turn, is any behavior within an intimate relationship that causes physical, psychological or sexual harm to those involved in the relationship. More specifically, IPV encompasses acts of physical, sexual and emotional violence, as well as controlling behaviors, by a current or former intimate partner^(2)^.

The focus of this study is IPV, with an emphasis on violence against women, the most common manifestation of domestic violence. In this context, the main perpetrators are the so-called “intimate partners”, including husbands, fiancés, boyfriends or any man who shares an intimate emotional bond with women. Although the term “domestic” can refer to a variety of family interactions, the use of the term “domestic violence” is used to describe violence exercised by men against women^(3,4)^.

Gender-based violence is a recognized and discussed global public health and human rights challenge, with considerable female morbidity and mortality. However, the violations and abuses faced by sex workers are rarely incorporated into discussions^(5)^. In addition to suffering violence in their private lives, these women are susceptible to violence arising from the particularities of their occupation, which is perceived by society as illicit, culminating in stigmatization and sociopolitical marginalization^(5,6)^.

Sex work is considered a consensual and paid work activity, in which individuals, usually women, offer sexual services to their clients in exchange for income that enables their support and subsistence^(7,8)^.

Sexual services performed by women are permeated by stigmas and prejudices that perpetuate social inequities based on gender, race and class^(6,8)^. These inequities are often manifested in the form of discrimination, economic exploitation and violence, exacerbating the marginalization of these workers and limiting their access to resources and opportunities. In countries where this activity is not legalized, the patriarchal system reinforces sex workers’ invisibility and vulnerability^(9)^.

In 2002, Brazil recognized the work performed by sex workers as a profession, by including it in the *Classificação Brasileira de Ocupações* (CBO, Brazilian Classification of Occupations)^(10)^, established by the Ministry of Labor and Employment. However, the lack of regulation prevents sex workers from having their rights fully guaranteed, including health and fundamental rights^(11)^.

Recognizing sex work as an economic negotiation in which women exercise control over their activities and seek financial autonomy also considers the dynamics of power, resistance, and the intersection of factors such as gender, class, and race. In this way, sex work is not only seen as an economic activity, but also as a strategic choice within specific contexts of social and financial inequality^(7)^.

Violence directed at sex workers can be linked to violence against women. The reasons that trigger violence are complex and multifaceted, and may be related to sexual involvement with other men in order to provide for the family^(5)^. Offenders are most often a male with whom the woman shares feelings, ideas and has a family bond^(12,13)^.

This traumatic experience causes significant changes in women’s lifestyles, weakening their self-confidence and generating a persistent distrust of people and new relationships. Many experience feelings of sadness, discouragement, loneliness and stress. Others, however, are able to face adversity and find ways to deal with the trauma, seek support, strengthen their self-esteem and gradually rebuild their lives^(12)^.

From this perspective, it is crucial to understand how these women develop their coping strategies when faced with adversity. The concept of coping refers to the processes and strategies that individuals use to deal with stressors that affect their state of balance. These processes involve cognitive and behavioral efforts to manage internal and external demands that are perceived as exceeding available personal resources^(14)^.

It is notable that women need protection in emotional, domestic and family contexts, regardless of their occupation or social status^(13)^. The *Maria da Penha* Law^(15)^, in Brazil, internationally recognized as one of the three most comprehensive and well-designed laws in the world regarding protection against violence directed at women, is an example of an important initiative in this regard, but protecting and combating violence against women are ongoing challenges that require constant efforts and awareness-raising in society^(16)^.

IPV against sex workers is a multifaceted issue rooted in unequal power dynamics. Recognizing and addressing this violence requires a holistic understanding that encompasses both the particularities faced by these women in their professional sphere and the personal vulnerabilities that permeate their private lives. Deepening knowledge in this field is crucial to formulating public policies and intervention strategies that not only combat violence, but also promote these workers’ health, safety and well-being, ensuring their inalienable right to dignity and physical and emotional integrity. Thus, the guiding question was: what are the narratives of sex workers about violence suffered by intimate partners and their coping strategies?

## OBJECTIVES

To understand the narratives of sex workers about violence suffered by intimate partners and their coping strategies.

## METHODS

### Ethical aspects

The research was approved by the *Universidade Federal de Pelotas* (UFPEL) School of Nursing Research Ethics Committee. The ethical precepts contained in Resolution 466/2012^(17)^ were followed at all stages of the research development. Participants were informed about the objectives and methodological procedures and signed the Informed Consent Form (ICF). Participant identity was preserved during data analysis and dissemination of results, using fictitious names chosen by the participants themselves, followed by a number corresponding to their age.

### Study design and theoretical-methodological framework

This is an excerpt from a master’s dissertation entitled “*Memórias de um passado presente: mulheres profissionais do sexo em tempos de pandemia do novo coronavírus 2019*”, presented to the Graduate Program in Nursing at UFPEL.

It is characterized by a qualitative approach research centered on the oral history method in the thematic modality. Oral history presents itself as a possibility for carrying out studies that aim to know people’s lives in depth, through the narrative of their memories^(18)^.

In writing this study, the COnsolidated criteria for REporting Qualitative research (COREQ) criteria were applied^(19)^.

### Study setting

The research was carried out in two houses, interviewees’ workplace, located in the center of a medium-sized city located in southern Brazil.

### Data source

Six women linked to a Non-Governmental Organization (NGO) participated in the study using the snowball technique, used in qualitative research to identify and recruit participants through a non-probabilistic sample, and its use is indicated in research when the population of interest is difficult to reach^(20)^.

The NGO facilitated the approach by indicating one of its participants with the greatest connection to the NGO to begin the interviews. Sex workers, 18 years old or older, with time available to respond to the interview and a connection with/being assisted by the NGO, were included. Sex workers that did not feel in good physical and/or mental health to interact with the researcher and respond to the survey were excluded.

### Data collection and organization

Ten women who met the inclusion criteria were invited to participate in the study, of which two refused and two were unable to respond to the interviews within the timetable stipulated for data collection, leaving six who responded to the interviews.

Data collection took place in March and April 2022, over the course of six visits. The interviews took place individually in a private environment, ensuring participant privacy. An oral history interview script was used as a flexible guide to guide the interaction between the researcher and the narrators. This script was divided into an initial section, focused on participant sociodemographic characterization, followed by a series of open-ended questions designed to identify the narratives^(18)^.

Prior contact was made, and interviews were scheduled through the instant messaging app WhatsApp^®^, according to each participant’s availability, and were conducted by a nurse, the study’s lead researcher, who had been previously trained and had experience in data collection. The guiding question that generated the narratives about violence perpetrated by intimate partners was “Tell me a little about your life story”, followed, when necessary, by the prompt “Have you ever experienced situations of violence?”.

The narratives were recorded in audio on a smartphone and lasted an average of 40 minutes, varying in a time interval between 32 and 51 minutes. The audios were transcribed in full in Microsoft Word 2010^®^ and stored on a portable storage device (flash drive), which was in the possession of the main researcher.

### Data analysis

The interview content was subjected to thematic content analysis^(21)^, following the stages: 11) pre-analysis - involved the full transcription of narratives in the temporal order of the interviews. Subsequently, a skim reading was carried out for an initial contact with the data and perception of the elements to be analyzed, followed by an exhaustive and careful reading of the material; 2) material exploration - coding was carried out, based on the cutout regarding the theme and numbered according to the frequency and intensity of the narratives; 3) data treatment - semantic and lexical categorization was carried out, taking into account the meanings and senses of the discourses^(21)^, based on which the following categories emerged: The nature of violence present in sex workers’ intimate relationships; Social circle and affective relationships - coping strategies.

## RESULTS

The study involved the participation of six cisgender women who were sex workers. Participants’ ages ranged from 34 to 53 years old, and the majority were born in the state of Rio Grande do Sul, with the exception of one, who was born in northeastern Brazil. Three of the participants identified themselves as white, whereas the other three identified themselves as black. Most of participants had children, and only one of them was not a mother. Regarding religiosity and/or religious practices, two participants reported not having any religious beliefs. All of them completed high school, two had technical training, and one was studying higher education. With the exception of one, all were separated. Half of them lived in their own homes, whereas the others lived in rented properties. Job tenure as sex workers ranged from three months to 38 years, and their monthly income ranged from R$2.000.00 (equivalent to US$363.63) to R$12.000.00 (equivalent to US$2,181.81).

From the data analysis process, two empirical categories were identified and organized.

### The nature of violence present in sex workers’ intimate relationships

Sex workers, as women, are also impacted by gender-based violence, with violence perpetrated by their intimate partners (spouses) being particularly notable in this group. The narratives help to elucidate the variety of violence experienced by these women in their life contexts and relationships, highlighting that psychological violence is the most prevalent.

*I was working as a prostitute and I met this father of my daughter. I lived with him for 17 years, a very abusive marriage, he never hit me, but psychologically yes, because he knew what I did* [prostitute] […]. (Luísa, 53)

*But my marriage was really complicated, I stayed in the marriage for convenience, for custom, because of my daughters, because nothing was good, the marriage wasn’t good, the sex was bad, he was jealous, I suffered threats, but I went, I stayed, until one day I took it and decided.* (Kaká, 42)

Among the women studied, psychological violence manifested itself through humiliation, verbal abuse, and excessive jealousy, creating an environment of emotional imprisonment and psychological exhaustion. The violence manifested in these women’s intimate relationships often does not occur in isolation, but rather in an interconnected manner, in which one form of violence may overlap or coexist with another.

*He was very aggressive, my last husband, from a marriage that lasted 16 years. He once attacked me* [...] *my ex-husband was very sexist, he is the type of man who has to have things his way, because he is the head of the house - I am the man, I am the one in charge, you understand?* (Kaká, 42)

*My marriage was very difficult. He didn’t accept the separation, he was aggressive, he hit me, he tried to kill me and it wasn’t just once, it was three times, he is registered with Maria da Penha. I had to register him there, so for me to be able to have my independence without needing him anymore, I chose this profession at the time because it was the most plausible one, which would have a faster income.* (Mari, 48)

The narratives presented outline the harsh reality of physical violence, attempted femicide, and authoritarian control in intimate relationships. The search for legal protection and financial independence, as a reaction to this violence, is mentioned as a means of escaping the abusive relationship. On the other hand, work, which often serves as a vehicle for independence, enabling women to escape violent relationships, can paradoxically become a factor of imprisonment.

[...] *my husband got involved with drugs and took everything I had, I worked here and left there, I didn’t have a decent pair of flip-flops to put on my feet, he spent everything I had.* (Paula, 38)

*Because our relationship was extremely possessive, he either wanted both* [mother and son] *or nothing. As he pursued me a lot, he didn’t want to separate and so on, I decided to completely cut ties.* (Brenda, 34)

In some circumstances, the same occupation that provides economic autonomy and a means of escaping abusive relationships can also be a place where partners are financially exploited. This duality reveals a complex and challenging scenario, in which the search for financial autonomy and personal security can be accompanied by new forms of vulnerability and exploitation. As a result, some people end up creating self-defense mechanisms, which often involve giving up the opportunity to establish new emotional ties.

### Social circle and affective relationships - coping strategies

Previous experiences of violence can have a profound impact on these women’s affective relationships, prompting them to adopt strategies for their protection. Previous experiences play a crucial role in shaping future perspectives and decisions, providing a knowledge base and reference from which they can operate, as can be seen in the following statements:

[...] *for me, having a romantic relationship, a boyfriend or anything else, my work gets in the way. So, I have never had a romantic relationship since I started working, almost 12 years ago, since I separated from my daughter’s father,* [...] *I preferred to choose my work, and since I can’t stop working now,* [...] *I will remain single* [...]. (Mari, 48)

*Today, I don’t have a relationship with anyone who knows what I do. If they know what I do, I don’t want it, because, in my head, if they know what I do, oh, you’re looking at so-and-so, you want so-and-so, or you’ve already given it to that one, are you going to leave, how much are you going to charge? We’re short on money here, so you’re going to give it to them on the corner, so that’s an abuse, you know? But having a relationship with someone who knows what I do, no, the money I earn is mine alone, an investment just for me.* (Luísa, 53)

[...] *I learned by seeing what happened, and I didn’t want it for myself, so I saw a girl being exposed, being manipulated, being extorted, blackmailed for what she did with her life, and that life was hers, I couldn’t give that right to someone else, so I don’t give that right to someone else.* (Paula, 38)

The narratives highlight the complex interaction between work and personal life of these women. Work is seen as a barrier to romantic relationships, and there is fear of exploitation or manipulation if a partner becomes aware. This leads to a clear separation between professional and personal life. However, one of them, who has no history of IPV, mentioned having been in a stable relationship for over six years, where her work is not an obstacle to a healthy relationship.

*Because when I understand that the people I relate to are becoming more private, becoming something more intimate, I tell them* […] *my private life has nothing to do with my life here, I leave here and have another life with my family, with my husband.* (Paula, 38)

The interlocutors reveal a notable distinction in the treatment given to them by their intimate partners in contrast to their clients, indicating that, for these workers, violence was prevalent in intimate relationships, but notably absent in interactions with clients. They also highlight important aspects that express possibilities for coping and support for different life situations.

*Only domestic violence. Violence, like, with a client wanting to hit me, never, quite the opposite, if I turned a foot, they would carry me in their arms, take me to the doctor, pay for rent, food. I worked for ten years in nightclubs, traveling almost all over Brazil, Rio, São Paulo. I worked in Santa Catarina, there was never any type of violence, only attention and affection.* (Luísa, 53)

*From clients, I have never, in fact, only been attacked by my husband. The clients have always treated me very well* [...]. (Mari, 48)

*I’ve never had any problems with clients, or being abused,* [...] *I’ve never been mistreated. Girls tell absurd things, but I’ve never been through that.* (Paula, 38)

*Thank God, I only have excellent clients, I have never experienced any act of violence, but I have seen colleagues suffering* [...]. (Brenda, 34)

The absence of violence by clients can be interpreted as an indication of respect and support, even though the perception of the risk inherent in the exercise of the profession remains latent. Although the profession offers relatively stable financial security and personal protection, this benefit contrasts with the reality of violence and abuse in the domestic environment, often resulting in weakened social and affective relationships.

## DISCUSSION

The results of this research reveal the occurrence of more than one type of violence. Among them, the most cited were verbal and psychological violence, followed by patrimonial and physical violence, all perpetrated by the intimate partner. There were no reports of work-related violence with clients.

A survey conducted in ten Brazilian cities revealed that the prevalence of verbal violence against female sex workers is 59.5%. When violence is committed by an intimate partner, this rate reaches 25.2%^(5)^. In Piauí, a study found that 60.5% of prostitutes interviewed experienced psychological violence, with 54.5% being victims of ex-boyfriends or boyfriends, and 90.9% of offenders being men^(22)^. A survey using an online platform showed that psychological violence prevailed in 60.58% of the responses obtained^(23)^.

Sex workers are entangled in a historical matrix of stigmatization and violence. The literature reports a range of violence suffered by these women, ranging from verbal assaults to homicide. Despite legislative efforts and civil activism to criminalize such violence, daily practice reveals the persistence of such oppressive dynamics. Occupational discrimination, combined with precarious socioeconomic conditions, exposes these women to increased vulnerability, subjecting them to abuse both in their professional and domestic lives by romantic partners and family members^(5)^.

A case study investigated 12 femicides of sex workers, identified in 94 police investigations into the murders of women in Porto Alegre between 2006 and 2010. Many of these cases involved intimate partners, such as ex-boyfriends, husbands or boyfriends. The deaths were more frequent than those of other women, highlighting the intersection between poverty, prostitution and abusive relationships, demonstrating these women’s vulnerability and the impact of misogyny in a patriarchal society^(24)^.

Social and economic vulnerability reinforces Brazilian women’s emotional and financial dependence on their partners. Thus, the intersectionality of gender, race and class demonstrates how these factors combine to increase sex workers’ exposure to violence^(24)^.

The idea of possession established in an intimate relationship denotes an escalation in violence that often begins verbally and gradually takes on more cruel forms, such as the risk of death. Thus, the importance of establishing a support network and implementing public policies aimed at protecting the lives of these women is emphasized^(25)^, as provided for in specific legislation to combat domestic and family violence^(15)^.

Women, in different situations, need protection in different environments, be they domestic, emotional, family and/or social. This highlights the use of this legislation as a protection device. The essay that reflects on this law highlights its importance, recognized internationally, characterized by its comprehensiveness and careful elaboration both in combating violence and in protecting women^(13,16)^.

Psychological violence can often be more subtle and less explicit than other forms of violence^(3,25)^. The results of this study showed that women were subjected to humiliation, degradation, threats, insults, comparisons and irony. Verbal violence, among other consequences, can end up preventing women from leaving the house, working, wearing certain clothes, studying and even expressing themselves. According to the Ministry of Health^(26)^, violence has serious impacts on victims’ mental and physical health, with emphasis on feelings of fear, sadness, sleep problems, depression and anxiety.

Ongoing psychological violence, in addition to causing emotional damage^(5)^, can also be responsible for apathy and immobility in affective relationships, with women often remaining in relationships despite the aggression imposed by their intimate partners.

The difficulty of separating from intimate partners, even after previous experiences of violence, is supported by research conducted in India, where sex workers’ relationships are often marked by feelings of fear and a sense of entrapment, with many of these women feeling that they cannot leave their partners. This scenario indicates a complex and difficult relationship, in which financial and social dependencies coexist with emotional challenges and, potentially, abuse within the dynamics of intimate partnership^(27)^.

Furthermore, the invisibility of sex workers’ mental health stems from a dehumanizing logic, which assumes that paid sex-related activity is immoral, dirty and illicit. Although the professional activity has been recognized by CBO^(10)^, there is an urgent need for advances in health policies that understand the specificities of the work performed by these workers, without neglecting the different contexts of life as well as their health and well-being^(9)^.

It was also observed that IPV could influence the search for and establishment of future relationships. In this regard, the history of violence must be considered as a factor that could shape the choices and decisions of these women in relation to interpersonal relationships^(3)^.

Thus, the presence of several strategies for coping with violence stands out, predominantly based on individual resources, without the support of affective or social networks. The interlocutors express the devastating impact of violence on their dignity, well-being and safety. As a strategy, they mention avoiding affective and intimate relationships to prevent new situations of violence. Moreover, they choose not to have relationships with people who know their profession and emphasize the importance of establishing clear boundaries between work and family relationships.

Women who are victims of violence may use coping strategies to survive or overcome the episodes they have suffered and to identify, deal with and avoid new events. These attitudes are dynamic and vary according to individuals’ needs to avoid problems, seek support or actively face them. Thus, coping response is an intentional action directed at a perceived stressor, involving an emotional or behavioral reaction^(28)^.

The way in which abused women seek to cope with these episodes is related to their experiences, personal characteristics, beliefs and values, and is influenced by access to social and material networks^(28)^. Thus, with knowledge of the coping strategies of victims of violence, support networks have great potential in reducing and combating aggression.

However, there is a significant gap in education that allows women to identify the types of violence and learn about the forms of protection they can access^(23)^. It is important to note that awareness of their rights and a structured network of health and social protection services are fundamental conditions for promoting the health of sex workers who are victims of IPV.

There is a shortage of Brazilian studies that address gender-based violence that these women suffer^(29)^. Hence, research that is guided by a holistic approach to health will be more connected with the complexity and multiple dimensions of these women’s lives, including reflecting on how experiences of violence impact their lives and future choices, also contributing to expanding this knowledge by considering the different contexts in which violence can occur.

### Study limitations

As a limitation, it is worth noting that the interviews were conducted with sex workers from only two groups, which, together with the qualitative methodology adopted, prevents the generalization of results to other populations or contexts. In addition to this, the sensitivity of the topic may lead to underreporting of IPV, since fear, guilt and associated stigma may restrict the disclosure of these experiences. The scarcity of specific literature on violence practiced against these women by their intimate partners also limits the comparison of the results of this study with other research.

### Contributions to nursing

Research into IPV and coping strategies adopted by female sex workers offers a valuable contribution to nursing, expanding professionals’ understanding of these dynamics, contributing to empowerment of victims, breaking the cycle of suffering and combating violence. It helps professionals identify signs of violence, offer appropriate support and develop more effective care interventions. Improving research in this area is crucial and could result in significant improvements in sex workers’ quality of care and well-being in the context studied.

## FINAL CONSIDERATIONS

Sex workers were victims of verbal, psychological, financial and physical violence by intimate partners. In this specific group, work-related violence with clients was not an occurrence, differing from findings in other studies.

Previous experiences of violence result in coping strategies such as avoiding romantic relationships and establishing clear boundaries between professional and personal life. These choices may be related to both the history of violence they have suffered and the concern to avoid possible situations of exploitation by partners. These findings highlight the complexity of these women’s experiences and the importance of understanding the contextual factors that shape their lives and decisions, highlighting the urgent need for public policies that address their particularities and ensure effective protection, promoting their safety and well-being.
