# Supplementary material for: Narratives of sex workers: intimate partner violence and coping strategies
Source: Rev Bras Enferm. 2024 Dec 16;77(6):e20240180. doi: 10.1590/0034-7167-2024-0180 (PMC11654522; doi:10.1590/0034-7167-2024-0180)
Supplement: Translation

# Translation: Narrativas de trabalhadoras sexuais: violência por parceiro íntimo e estratégias de enfrentamento

**DOI:** 10.1590/0034-7167-2024-0180pt

**Authors:** Milena Oliveira Costa, Valéria Cristina Christello Coimbra, Michele Mandagará de Oliveira, Clarissa de Souza Cardoso, Vania Dias Cruz, Ariane da Cruz Guedes, Josiane da Costa Moreira, Luciano Santos Gentilini

**Affiliations:** I Universidade Federal de Pelotas. Pelotas, Rio Grande do Sul, Brasil; II Universidade Federal do Pampa. Uruguaiana, Rio Grande do Sul, Brasil

**Keywords:** Profissionais do Sexo, Violência por Parceiro Íntimo, Mulheres, Narrativa Pessoal, Estratégias de Enfrentamento.

## Abstract

**Objetivos:**

conhecer as narrativas de trabalhadoras sexuais acerca da violência sofrida por parceiros íntimos e as suas estratégias de enfrentamento.

**Métodos:**

pesquisa qualitativa, centrada na história oral temática, realizada com seis trabalhadoras sexuais no sul do Brasil, que responderam a entrevistas em profundidade através de um roteiro flexível. Foi utilizada a análise de conteúdo temática.

**Resultados:**

participaram do estudo mulheres cisgênero que se autodeclararam metade negras e metade brancas. A maioria tinha filhos e estava separada. Relataram relações abusivas por parte de seus parceiros íntimos, destacando-se a violência verbal, psicológica, patrimonial, física e tentativa de feminicídio. Tais violências resultaram em estratégias de enfrentamento, como evitar vínculos afetivos e manter uma vida discreta.

**Considerações Finais:**

a violência por parceiro íntimo é prevalente entre as participantes, levando-as a adotar estratégias para preservar sua segurança e bem-estar, evidenciando a necessidade de políticas públicas que atendam às suas particularidades e garantam proteção.

## INTRODUÇÃO

Conforme a definição da Organização das Nações Unidas, a violência contra as mulheres é descrita como “qualquer ato de violência de gênero que resulte ou possa resultar em danos ou sofrimentos físicos, sexuais ou mentais para as mulheres, inclusive ameaças de tais atos, coação ou privação arbitrária de liberdade, seja em vida pública ou privada”^(1)^.

A violência por parceiro íntimo (VPI), por sua vez, é qualquer comportamento dentro de uma relação íntima que cause dano físico, psicológico ou sexual aos envolvidos na relação. Mais especificamente, a VPI abrange atos de violência física, sexual e emocional, bem como comportamentos controladores, por um parceiro íntimo atual ou anterior^(2)^.

O foco deste estudo é a VPI, com ênfase na violência contra as mulheres, manifestação mais recorrente de violência doméstica. Nesse panorama, os principais perpetradores são os denominados “parceiros íntimos”, incluindo maridos, noivos, namorados ou qualquer homem que compartilhe um laço afetivo íntimo com a mulher. Embora o termo “doméstica” possa se referir a diversas interações familiares, o uso do termo “violência doméstica” é empregado para descrever a violência exercida por homens em desfavor das mulheres^(3,4)^.

A violência baseada em gênero é um reconhecido e discutido desafio global de saúde pública e direitos humanos, com considerável morbidade e mortalidade feminina. Entretanto, raramente são incorporados nas discussões as violações e os abusos enfrentados por trabalhadoras sexuais^(5)^. Além de sofrerem violência em suas vidas privadas, essas mulheres são suscetíveis à violência oriunda das particularidades de sua ocupação, percebida pela sociedade como ilícita, culminando em estigmatização e marginalização sociopolítica^(5,6)^.

Considera-se trabalho sexual uma atividade laboral consentida e remunerada, na qual indivíduos, geralmente mulheres, ofertam serviços sexuais a seus clientes em troca de renda que possibilite seu sustento e subsistência^(7,8)^.

O serviço sexual exercido por mulheres é permeado por estigmas e preconceitos que perpetuam iniquidades sociais de gênero, raça e classe^(6,8)^. Essas iniquidades são frequentemente manifestadas na forma de discriminação, exploração econômica e violência, exacerbando a marginalização dessas trabalhadoras e limitando o seu acesso a recursos e oportunidades. Em países onde essa atividade não é legalizada, o sistema patriarcal reforça a invisibilidade e vulnerabilidade das trabalhadoras sexuais^(9)^.

Em 2002, o Brasil reconheceu o trabalho realizado pelas trabalhadoras sexuais como profissão, ao incluí-lo na Classificação Brasileira de Ocupações (CBO)^(10)^, instituída pelo Ministério do Trabalho e Emprego. Contudo, a falta de regulamentação impede que as trabalhadoras sexuais tenham seus direitos plenamente assegurados, incluindo os de saúde e direitos fundamentais^(11)^.

Reconhecendo o trabalho sexual como uma negociação econômica, na qual as mulheres exercem controle sobre suas atividades e buscam autonomia financeira, consideram-se também as dinâmicas de poder, resistência e a interseção de fatores como gênero, classe e raça. Dessa forma, o trabalho sexual não é visto apenas como uma atividade econômica, mas também como uma escolha estratégica dentro de contextos específicos de desigualdade social e financeira^(7)^.

A violência dirigida às trabalhadoras sexuais pode ser vinculada à violência contra as mulheres. Os motivos que desencadeiam a violência são complexos e multifacetados, podendo estar relacionados ao envolvimento sexual com outros homens, a fim de prover o sustento da família^(5)^. O agressor, na maioria das vezes, é alguém do sexo masculino, com quem a mulher compartilha sentimentos, ideias e tem vínculo familiar^(12,13)^.

Essa vivência traumática acarreta mudanças significativas no estilo de vida das mulheres, enfraquecendo sua autoconfiança e gerando uma persistente desconfiança nas pessoas e em novos relacionamentos. Muitas apresentam sentimentos de tristeza, desânimo, solidão e estresse. Já outras apresentam capacidade de enfrentar as adversidades, e encontram maneiras de lidar com o trauma, buscar apoio, fortalecer sua autoestima e, gradualmente, reconstruir suas vidas^(12)^.

Nessa perspectiva, torna-se crucial entender como essas mulheres desenvolvem suas estratégias de enfrentamento/*coping* diante das adversidades. O conceito de *coping* refere-se aos processos e estratégias que os indivíduos utilizam para lidar com estressores que afetam seu estado de equilíbrio. Esses processos envolvem esforços cognitivos e comportamentais para gerenciar as demandas internas e externas que são percebidas como excedendo os recursos pessoais disponíveis^(14)^.

É notável que as mulheres necessitam de proteção nos contextos afetivo, doméstico e familiar, independentemente de sua ocupação ou *status* social^(13)^. A Lei Maria da Penha^(15)^, no Brasil, reconhecida internacionalmente como uma das três legislações mais abrangentes e bem elaboradas do mundo no que se refere à proteção contra a violência direcionada às mulheres, é um exemplo de iniciativa importante nesse sentido, mas a proteção e o combate à violência contra as mulheres são desafios contínuos que exigem esforços constantes e a conscientização da sociedade^(16)^.

A VPI contra trabalhadoras sexuais constitui uma questão multifacetada e enraizada em dinâmicas de poder desiguais. Reconhecer e abordar essa violência requer uma compreensão holística que engloba tanto as particularidades enfrentadas por essas mulheres em sua esfera profissional quanto as vulnerabilidades pessoais que permeiam suas vidas privadas. O aprofundamento do conhecimento nesse campo é crucial para formular políticas públicas e estratégias de intervenção que não somente combatam a violência, mas também promovam a saúde, a segurança e o bem-estar dessas trabalhadoras, assegurando seu direito inalienável à dignidade e à integridade física e emocional. Assim, traçou-se como questão norteadora: quais as narrativas de trabalhadoras sexuais acerca da violência sofrida por parceiros íntimos e as suas estratégias de enfrentamento?

## OBJETIVOS

Conhecer as narrativas de trabalhadoras sexuais acerca da violência sofrida por parceiros íntimos e as suas estratégias de enfrentamento.

## MÉTODOS

### Aspectos éticos

A pesquisa foi aprovada pelo Comitê de Ética em Pesquisa com Seres Humanos da Faculdade de Enfermagem da Universidade Federal de Pelotas (UFPEL). Em todas as etapas do desenvolvimento da pesquisa, foram cumpridos os preceitos éticos contidos na Resolução nº. 466/2012^(17)^. As participantes foram esclarecidas quanto aos objetivos e procedimentos metodológicos, e assinaram o Termo de Consentimento Livre e Esclarecido (TCLE). A identidade das participantes do estudo foi preservada durante a análise dos dados e divulgação dos resultados, utilizando nomes fictícios escolhidos por elas próprias, seguido do número correspondente à idade.

### Tipo de estudo e referencial teórico-metodológico

Trata-se de recorte da dissertação de mestrado intitulada “Memórias de um passado presente: mulheres profissionais do sexo em tempos de pandemia do novo coronavírus 2019”, apresentada ao Programa de Pós-Graduação em Enfermagem da UFPEL.

Caracteriza-se por uma pesquisa de abordagem qualitativa centrada no método da história oral na modalidade temática. A história oral apresenta-se como uma possibilidade para realização de estudos que visem conhecer profundamente a vida das pessoas, através da narrativa de suas memórias^(18)^.

Na redação deste estudo aplicaram-se os critérios apresentados pelo guia *COnsolidated criteria for REporting Qualitative research* (COREQ)^(19)^.

### Cenário do estudo

A pesquisa foi realizada em duas casas, local de trabalho das entrevistadas, situadas no centro de uma cidade de médio porte localizada no sul do Brasil.

### Fonte de dados

Participaram do estudo seis mulheres vinculadas a uma Organização Não Governamental (ONG), através da técnica *snowball*, usada em pesquisas qualitativas para identificar e recrutar participantes através de uma amostra não probabilística, sendo seu uso indicado em pesquisas quando a população de interesse é difícil de alcançar^(20)^.

A ONG propiciou a aproximação, indicando uma de suas participantes com maior vínculo para o início das entrevistas. Os critérios de inclusão foram estar atuando como trabalhadora sexual, ter idade igual ou superior a 18 anos, disponibilidade de tempo para responder à entrevista e possuir vínculo/ser assistida pela ONG. O critério de exclusão foi não se sentir em condições de saúde física e/ou mental para interagir com a pesquisadora e responder à pesquisa.

### Coleta e organização dos dados

Foram convidadas a participar do estudo dez mulheres que atendiam aos critérios de inclusão; dessas, duas recusaram e duas não conseguiram responder às entrevistas dentro do cronograma estipulado para a coleta de dados, permanecendo seis, que responderam às entrevistas.

A coleta de dados ocorreu nos meses de março e abril de 2022, ao longo de seis visitas. As entrevistas ocorreram de forma individual em ambiente reservado, assegurando a privacidade das participantes. Utilizou-se um roteiro de entrevista em história oral como um guia flexível para conduzir a interação entre a pesquisadora e as narradoras. Esse roteiro foi dividido em uma seção inicial focada na caracterização sociodemográfica das participantes, seguida por uma série de questões abertas projetadas para captação das narrativas^(18)^.

Foi realizado contato prévio, e as entrevistas foram agendadas por meio do aplicativo de conversa instantânea *WhatsApp^®^*, de acordo com a disponibilidade de cada participante, sendo conduzidas por uma enfermeira, pesquisadora principal do estudo, previamente capacitada e com experiência em coleta de dados. A questão norteadora que gerou as narrativas sobre a violência perpetrada por parceiros íntimos foi “Conte-me um pouco da sua história de vida”, seguida, quando necessário, do estímulo “Você já passou por situações de violência?”.

As narrativas foram gravadas em áudio por um *smartphone,* e tiveram duração média de 40 minutos, variando em um intervalo de tempo entre 32 e 51 minutos. Os áudios foram transcritos na íntegra no *software Microsoft Word* 2010^®^ e armazenados em um dispositivo de armazenamento portátil (*pendrive*), estando esse em poder da pesquisadora principal.

### Análise dos dados

O conteúdo das entrevistas foi submetido à análise de conteúdo temática^(21)^, seguindo as etapas de: 1) pré-análise - envolveu a transcrição integral das narrativas em ordem temporal das entrevistas. Posteriormente, foi realizada uma leitura flutuante para um contato inicial com os dados e percepção dos elementos a serem analisados, seguida por uma leitura exaustiva e criteriosa do material; 2) exploração do material - procedeu-se à codificação, baseada no recorte quanto ao tema e numerada de acordo com a frequência e intensidade das narrativas; 3) tratamento dos dados - efetuou-se a categorização semântica e léxica, levando em conta os significados e sentidos dos discursos^(21)^, com base nos quais emergiram as categorias: A natureza das violências presentes nas relações íntimas das trabalhadoras sexuais; Círculo social e relacionamentos afetivos - estratégias de enfrentamento.

## RESULTADOS

A pesquisa envolveu a participação de seis mulheres cisgênero trabalhadoras sexuais. A faixa etária das participantes variou entre 34 e 53 anos, sendo a maioria nascida no estado do Rio Grande do Sul, com exceção de uma, natural da região Nordeste do Brasil. Três das participantes se autodeclararam de etnia branca, enquanto que as outras três se identificaram como negras. A maioria das participantes tinha filhos, sendo que apenas uma delas não era mãe. No que diz respeito à religiosidade e/ou práticas religiosas, duas participantes referiram não ser adeptas a nenhuma crença religiosa. Todas concluíram o ensino médio, sendo que duas possuíam formação técnica e uma estava cursando o ensino superior. Com exceção de uma, todas eram separadas. Metade delas residia em casa própria, enquanto as demais viviam em imóveis alugados. Quanto ao tempo de atuação como trabalhadoras sexuais, variou de três meses a 38 anos, e a renda mensal variou de R$ 2.000,00 a R$ 12.000,00.

A partir do processo de análise dos dados, foram identificadas e organizadas duas categorias empíricas.

### A natureza das violências presentes nas relações íntimas das trabalhadoras sexuais

A trabalhadora sexual, na sua qualidade de mulher, também é impactada pela violência de gênero, sendo notável neste grupo a violência perpetrada por seus parceiros íntimos (cônjuges). As narrativas contribuem para elucidar a variedade de violências vivenciadas pelas mesmas em seus contextos de vida e relacionamentos, ressaltando que a violência psicológica é a que prevalece.

*Eu estava fazendo programa e conheci esse pai da minha filha. Vivi com ele 17 anos, um casamento muito abusivo, nunca me bateu, mas psicologicamente sim, porque ele sabia o que eu fazia* [programa] […]. (Luísa, 53)

*Mas foi bem complicado mesmo o meu casamento, eu me mantive no casamento mesmo por comodidade, por costume, por causa das minhas filhas, porque nada era bom, o casamento não era bom, o sexo era ruim, ele era ciumento, eu sofria ameaças, mas fui, me mantive, até que um dia eu peguei e decidi.* (Kaká, 42)

Entre as mulheres estudadas, a violência psicológica manifestou-se através de humilhação, violência verbal e ciúme excessivo, criando um ambiente de aprisionamento emocional e desgaste psicológico. As violências manifestadas nos relacionamentos íntimos dessas mulheres muitas vezes não ocorrem isoladamente, mas sim de forma interligada, em que uma forma de violência pode se sobrepor ou coexistir com outra.

*Ele era bem agressivo, meu último marido, do casamento que durou 16 anos. Uma vez ele me agrediu* [...] *meu ex-marido era muito machista, ele é do tipo de homem que as coisas têm que ser do jeito dele, porque ele é o chefe da casa - eu sou o homem, sou eu que mando, entendeu?* (Kaká, 42)

*Meu casamento era muito difícil. Ele não aceitava a separação, era agressivo, ele me batia, ele tentou me matar e não foi uma vez só, foram três vezes, ele está registrado na Maria da Penha. Eu tive que registrar ele lá, então, pra mim poder ter a minha independência sem precisar mais dele, eu optei por essa profissão na época, que foi o que era mais plausível, que teria um ganho de dinheiro mais rápido.* (Mari, 48)

As narrativas apresentadas delineiam a dura realidade da violência física, tentativa de feminicídio e do controle autoritário nas relações íntimas. A busca por proteção legal e independência financeira, como reação a essa violência, é mencionada como um meio de escapar da relação abusiva. Por outro lado, o trabalho, que muitas vezes serve como um veículo de independência, possibilitando às mulheres escaparem de relações violentas, pode paradoxalmente tornar-se um fator de aprisionamento.

[...] *meu marido se envolveu com droga e tirava tudo que eu tinha, eu trabalhava aqui e ia embora ali, não tinha um chinelo decente para botar no pé, ele gastava tudo que eu tinha.* (Paula, 38)

*Porque, assim, o nosso relacionamento, ele era extremamente possessivo, tanto ele queria ou os dois* [mãe e filho] *ou nada. Como ele me perseguia muito, não queria separar e tal, decidi cortar totalmente os vínculos.* (Brenda, 34)

Em algumas circunstâncias, a mesma ocupação, que proporciona autonomia econômica e um meio de fuga de relações abusivas, pode também ser o campo onde ocorre a exploração financeira por parte de parceiros. Essa dualidade revela um cenário intricado e desafiador, no qual a busca por autonomia financeira e segurança pessoal pode ser acompanhada de novas formas de vulnerabilidade e exploração. Em virtude disso, algumas acabam por criar mecanismos de autodefesa, os quais frequentemente implicam a renúncia à oportunidade de estabelecer novos laços afetivos.

### Círculo social e relacionamentos afetivos - estratégias de enfrentamento

As vivências anteriores de violência podem ter um impacto profundo nas relações afetivas dessas mulheres, impulsionando-as a adotar estratégias para sua proteção. As experiências prévias desempenham um papel crucial na moldagem das perspectivas e decisões futuras, oferecendo uma base de conhecimento e referência a partir da qual podem operar, conforme percebe-se nas falas a seguir:

[...] *para mim, ter um relacionamento afetivo, um namorado ou qualquer coisa que seja, o meu trabalho atrapalha. Então, eu nunca mais tive relacionamento afetivo desde que comecei a trabalhar, quase 12 anos pra cá, desde que eu separei do pai da minha filha,* [...] *preferi optar pelo meu trabalho, e como eu não posso parar de trabalhar agora,* [...] *vou continuar solteira* [...]. (Mari, 48)

*Hoje, eu não tenho relacionamento com ninguém que saiba o que eu faço. Se souber o que eu faço, eu não quero, porque, dentro da minha cabeça, se souber o que eu faço, ah, tu está olhando fulano, tu quer fulano, ou tu já deu para aquele, tu vai sair, tu vai cobrar quanto? Estamos ruim aqui de dinheiro, então tu vai dá ali na esquina, então esse é um abuso, sabe? Mas me relacionar com alguém que saiba o que eu faço, não, o dinheiro que eu ganho é só meu, investimento só para mim.* (Luísa, 53)

[...] *eu aprendi vendo o que acontecia, e eu não queria para mim, então eu vi menina sendo exposta, sendo manipulada, sendo extorquida, chantageada pelo que ela fazia da vida dela, e era dela aquela vida, não poderia dar esse direito a outra pessoa, então eu não dou esse direito à outra pessoa.* (Paula, 38)

As narrativas destacam a interação complexa entre o trabalho e a vida pessoal dessas mulheres. O trabalho é visto como barreira para relacionamentos afetivos, e há receio de exploração ou manipulação se o parceiro tiver conhecimento. Isso leva a uma separação clara entre vida profissional e pessoal. No entanto, uma delas, que não possui histórico de VPI, mencionou possuir uma relação estável há mais de seis anos, na qual o seu trabalho não é um empecilho para uma relação saudável.

*Porque as pessoas que eu me relaciono, quando entendo que aquela relação está ficando mais particular, virando uma coisa mais íntima, eu conto* […] *a minha vida privada não tem nada a ver com a minha vida aqui. Saio daqui da porta e tenho outra vida com a minha família, com meu marido.* (Paula, 38)

As interlocutoras revelam uma distinção notável no tratamento dispensado a elas por seus parceiros íntimos, em contraste com seus clientes, indicando que, para essas trabalhadoras, a violência era prevalente em relações íntimas, mas notavelmente ausente nas interações com clientes. Também salientam aspectos importantes que exprimem possibilidades de enfrentamento e apoio para as diversas situações de vida.

*Violência só doméstica. Violência, assim, com cliente querer bater, nunca, bem pelo contrário, se eu virasse um pé, queriam me carregar no colo, me levar no médico, pagar aluguel, comida. Trabalhei dez anos em boates, viajando quase todo o Brasil, Rio, São Paulo. Trabalhei em Santa Catarina, nunca teve nenhum tipo de violência, só atenção e carinho.* (Luísa, 53)

*Dos clientes, nunca, na realidade, só fui agredida pelo meu marido. Os clientes sempre me trataram super bem* [...]. (Mari, 48)

*Nunca tive problema com cliente, essas coisas de ser violentada,* [...] *eu nunca fui maltratada. As gurias contam coisas absurdas, eu nunca passei por isso.* (Paula, 38)

*Graças a Deus, eu tenho só clientes excelentes, eu nunca passei por nenhum ato de violência, mas eu já presenciei colegas* [...]. (Brenda, 34).

A ausência de violência por parte de clientes pode ser interpretada como um indicativo de respeito e amparo, ainda que a percepção do risco inerente ao exercício da profissão permaneça latente. Ainda que a profissão ofereça uma segurança financeira e uma proteção pessoal relativamente estáveis, esse benefício se contrapõe à realidade da violência e do abuso em âmbito doméstico, resultando frequentemente em relações sociais e afetivas fragilizadas.

## DISCUSSÃO

Os resultados da presente pesquisa denunciam a ocorrência de mais de um tipo de violência. Entre elas, as mais citadas foram a violência verbal e a violência psicológica, seguidas da violência patrimonial e violência física, todas praticadas pelo parceiro íntimo. Não houve relato de violência relacionada ao trabalho com clientes.

Pesquisa realizada em dez cidades brasileiras revelou que a prevalência de violência verbal contra mulheres trabalhadoras do sexo é de 59,5%. Quando a violência é cometida por um parceiro íntimo, essa taxa chega a 25,2%^(5)^. No Piauí, estudo identificou que 60,5% das prostitutas entrevistadas experienciaram violência psicológica, com 54,5% sendo vítimas de ex-namorados ou namorados, e 90,9%, dos agressores sendo homens^(22)^. Levantamento, por meio de uma plataforma *online*, evidenciou que a violência psicológica prevaleceu em 60,58% das respostas obtidas^(23)^.

As trabalhadoras sexuais estão imbricadas em uma matriz histórica de estigmatização e violência. A literatura reporta uma gama de violências sofridas por essas mulheres que se estende de agressões verbais a homicídios. A despeito dos esforços legislativos e ativismo civil para a criminalização dessas violências, a prática cotidiana revela a persistência de tais dinâmicas opressivas. A discriminação ocupacional, aliada a condições socioeconômicas precárias, expõe essas mulheres a uma vulnerabilidade acrescida, sujeitando-as a abusos, tanto no exercício profissional quanto no domínio doméstico, por parceiros afetivos e familiares^(5)^.

Estudo de caso investigou 12 feminicídios de trabalhadoras sexuais, identificados em 94 inquéritos policiais de assassinatos de mulheres em Porto Alegre entre 2006 e 2010. Muitos desses casos envolvem parceiros íntimos, como ex-namorados, maridos ou namorados. As mortes apresentaram uma frequência elevada em relação a outras mulheres, evidenciando a interseção entre pobreza, prostituição e relações abusivas, demonstrando a vulnerabilidade dessas mulheres e o impacto da misoginia na sociedade patriarcal^(24)^.

A vulnerabilidade social e econômica reforça a dependência afetiva, emocional e financeira das mulheres brasileiras com relação a seus companheiros. Assim, a interseccionalidade de gênero, raça e classe demonstra como esses fatores se combinam para aumentar a exposição das trabalhadoras sexuais à violência^(24)^.

A ideia de posse estabelecida na relação íntima denota uma escalada na violência que, muitas vezes, inicia-se de forma verbal e vai assumindo contornos mais cruéis, como o risco de morte. Assim, enfatiza-se a importância de estabelecer uma rede de apoio e de efetivar políticas públicas destinadas à proteção da vida dessas mulheres^(25)^, como previsto na legislação específica de combate à violência doméstica e família^(15)^.

As mulheres, em diversas situações, necessitam de proteção em diferentes ambientes, sejam eles doméstico, afetivo, familiar e/ou social. Evidencia-se, assim, a utilização dessa legislação como dispositivo de proteção. No ensaio que reflete sobre essa lei, destaca-se a sua importância, reconhecida internacionalmente, caracterizando-se pela abrangência e elaboração criteriosa tanto no combate à violência quanto na proteção das mulheres^(13,16)^.

A violência psicológica pode, muitas vezes, ser mais sutil e menos explícita que as demais formas de violência^(3,25)^. Os resultados do estudo demonstram que as mulheres foram submetidas a humilhações, degradações, ameaças, insultos, comparações e ironias. A violência verbal, entre outras consequências, pode acabar impedindo as mulheres de sair de casa, trabalhar, vestir determinada roupa, estudar e até mesmo expressar-se. De acordo com o Ministério da Saúde^(26)^, a violência tem graves impactos sobre a saúde mental e física das vítimas, com destaque para sentimentos de medo, tristeza, problemas de sono, depressão e ansiedade.

A violência psicológica contínua, além de ocasionar danos emocionais^(5)^, também pode ser responsável pela apatia e imobilidade nas relações afetivas, sendo comum as mulheres se manterem nos relacionamentos mesmo com as agressões impostas por seus parceiros íntimos.

A dificuldade de se separar dos parceiros íntimos, mesmo após experiências prévias de violência, é corroborada por pesquisa realizada na Índia, onde as relações das trabalhadoras sexuais são, muitas vezes, marcadas por sentimentos de medo e uma sensação de aprisionamento, com muitas dessas mulheres sentindo que não podem deixar seus parceiros. Esse cenário indica uma relação complexa e difícil, no qual dependências financeira e social coexistem com desafios emocionais e, potencialmente, abusos dentro da dinâmica da parceria íntima^(27)^.

Outrossim, a invisibilidade sobre a saúde mental das trabalhadoras sexuais provém de uma lógica desumanizadora, a qual supõe que a atividade remunerada relacionada ao sexo é imoral, suja e ilícita. Ainda que a atividade profissional tenha sido reconhecida pelo CBO^(10)^, há uma urgente necessidade de avanços nas políticas de saúde que compreendam as especificidades do trabalho exercido por essas trabalhadoras, sem negligenciar os diferentes contextos de vida, bem como sua saúde e bem-estar^(9)^.

Observou-se, ainda, a possibilidade de a violência por parceiros íntimos influenciar a busca e o estabelecimento de relacionamentos futuros. Nesse sentido, há que se considerar o histórico de violência como um fator que poderá moldar as escolhas e decisões dessas mulheres em relação aos relacionamentos interpessoais^(3)^.

Assim, destaca-se a presença de diversas estratégias para o enfrentamento da violência, predominantemente baseadas em recursos individuais, sem o apoio de redes afetivas ou sociais. As interlocutoras expressam o impacto devastador da violência sobre suas dignidades, bem-estar e segurança. Como estratégia, mencionam evitar relacionamentos afetivos e íntimos para prevenir novas situações de violência. Além disso, escolhem não se relacionar com pessoas que conhecem sua profissão, e ressaltam a importância de estabelecer limites claros entre as relações de trabalho e familiares.

Mulheres vítimas de violência podem utilizar estratégias de enfrentamento para sobreviver ou superar os episódios sofridos e para identificar, lidar e evitar novos eventos. Essas atitudes são dinâmicas e variam conforme as necessidades dos indivíduos, para evitar problemas, buscar suporte ou enfrentá-los ativamente. Assim, a resposta de *coping* é uma ação intencional direcionada a um estressor percebido, envolvendo uma reação emocional ou comportamental^(28)^.

A maneira como as mulheres violentadas buscam enfrentar esses episódios está relacionada às suas experiências, características pessoais, crenças e valores, sendo influenciada pelo acesso às redes sociais e materiais^(28)^. Dessa forma, com o conhecimento das estratégias de enfrentamento das vítimas de violência, as redes de apoio apresentam grande potencial na redução e combate às agressões.

No entanto, há uma lacuna importante na educação que permite às mulheres identificar os tipos de violência e conhecer as formas de proteção a que podem ter acesso^(23)^. Ressalta-se que a consciência de seus direitos e uma rede estruturada de serviços de saúde e proteção social são condições fundamentais para a promoção da saúde das trabalhadoras sexuais vítimas de VPI.

Existe uma escassez de trabalhos brasileiros que abordem a violência de gênero que essas mulheres sofrem^(29)^. Nesse sentido, pesquisas que se orientem por uma abordagem holística da saúde estarão mais conectadas com a complexidade e múltiplas dimensões da vida dessas mulheres, inclusive refletindo sobre como as experiências de violência impactam suas vidas e escolhas futuras, contribuindo ainda para ampliar esse conhecimento, ao considerar os diferentes contextos nos quais a violência pode ocorrer.

### Limitações do estudo

Como limitação, destaca-se que as entrevistas foram conduzidas com trabalhadoras sexuais de apenas dois grupos, o que, juntamente com a metodologia qualitativa adotada, impede a generalização dos resultados para outras populações ou contextos. Além disso, a sensibilidade do tema pode acarretar subnotificação da VPI, já que o medo, a culpa e o estigma, associados, podem restringir a revelação dessas experiências. A escassez de literatura específica sobre a violência praticada contra essas mulheres por seus parceiros íntimos também limita a comparação dos resultados deste estudo com outras pesquisas.

### Contribuições para a área da enfermagem

A investigação da VPI e das estratégias de enfrentamento adotadas por mulheres trabalhadoras sexuais oferece uma valiosa contribuição para a enfermagem, ampliando a compreensão dos profissionais sobre essas dinâmicas, contribuindo para empoderamento das vítimas, ruptura do ciclo sofrido e combate à violência. Auxilia os profissionais a identificar sinais de violência, a oferecer suporte adequado e a desenvolver intervenções de cuidado mais eficazes. O aprimoramento da pesquisa nessa área é crucial, podendo resultar em melhorias significativas na qualidade do atendimento e no bem-estar das trabalhadoras sexuais diante do contexto estudado.

## CONSIDERAÇÕES FINAIS

As trabalhadoras sexuais foram vítimas de violência verbal, psicológica, patrimonial e física por parte de parceiros íntimos. Nesse grupo específico, a violência relacionada ao trabalho com os clientes não foi uma ocorrência, diferindo de constatações encontradas em outros estudos.

As experiências prévias de violência resultam em estratégias de enfrentamento, como evitar relacionamentos afetivos e estabelecer limites claros entre a vida profissional e a pessoal. Essas escolhas podem estar relacionadas tanto ao histórico de violência que sofreram quanto à preocupação em evitar possíveis situações de exploração por parte de parceiros. Esses achados destacam a complexidade das experiências dessas mulheres e a importância de compreender os fatores contextuais que moldam suas vidas e decisões, evidenciando a necessidade urgente de políticas públicas que atendam às suas particularidades e garantam proteção eficaz, promovendo sua segurança e bem-estar.
